# Analyses of erythropoiesis from embryonic stem cell‐CD34^+^ and cord blood‐CD34^+^ cells reveal mechanisms for defective expansion and enucleation of embryomic stem cell‐erythroid cells

**DOI:** 10.1111/jcmm.17263

**Published:** 2022-03-05

**Authors:** Shihui Wang, Huizhi Zhao, Huan Zhang, Chengjie Gao, Xinhua Guo, Lixiang Chen, Cheryl Lobo, Karina Yazdanbakhsh, Shijie Zhang, Xiuli An

**Affiliations:** ^1^ School of Life Sciences Zhengzhou University Zhengzhou China; ^2^ Laboratory of Membrane Biology New York Blood Center New York New York USA; ^3^ Laboratory of Blood Borne Parasites New York Blood Center New York New York USA; ^4^ Laboratory of Complement Biology New York Blood Center New York New York USA

**Keywords:** ATAC‐Seq, enucleation, erythropoiesis, RNA‐Seq

## Abstract

Red blood cells (RBCs) generated ex vivo have the potential to be used for transfusion. Human embryonic stem cells (ES) and induced pluripotent stem cells (iPS) possess unlimited self‐renewal capacity and are the preferred cell sources to be used for ex vivo RBC generation. However, their applications are hindered by the facts that the expansion of ES/iPS‐derived erythroid cells is limited and the enucleation of ES/iPS‐derived erythroblasts is low compared to that derived from cord blood (CB) or peripheral blood (PB). To address this, we sought to investigate the underlying mechanisms by comparing the in vitro erythropoiesis profiles of CB CD34^+^ and ES CD34^+^ cells. We found that the limited expansion of ES CD34^+^ cell‐derived erythroid cells was associated with defective cell cycle of erythroid progenitors. In exploring the cellular and molecular mechanisms for the impaired enucleation of ES CD34^+^ cell‐derived orthochromatic erythroblasts (ES‐ortho), we found the chromatin of ES‐ortho was less condensed than that of CB CD34^+^ cell‐derived orthochromatic erythroblasts (CB‐ortho). At the molecular level, both RNA‐seq and ATAC‐seq analyses revealed that pathways involved in chromatin modification were down‐regulated in ES‐ortho. Additionally, the expression levels of molecules known to play important role in chromatin condensation or/and enucleation were significantly lower in ES‐ortho compared to that in CB‐ortho. Together, our findings have uncovered mechanisms for the limited expansion and impaired enucleation of ES CD34^+^ cell‐derived erythroid cells and may help to improve ex vivo RBC production from stem cells.

## 1 INTRODUCTION

Blood transfusion is the first form of cell therapy explored beginning in the 17th century^1^ and is an indispensable part of modern medical practice. Red blood cell (RBC) transfusion is lifesaving for trauma patients; it permits implementation of many lifesaving surgeries; and it is the major therapeutic option for patients with anaemias such as thalassemia^2^ and sickle cell disease.^3^ It is also used as a supportive care for cancer treatments. Currently, blood products used for transfusion are obtained through volunteer blood donations by eligible donors. Although the blood supply is generally sufficient in developed countries except during selected times during the year, a significant problem that is becoming increasingly important at the present time is in providing appropriately antigen matched red cell products for chronically transfused patients who are allo‐immunized including sickle cell patients. There is an additional concern that with the ageing of society, declining birth rates and increases in infectious diseases, the number of people requiring blood transfusion will increase while the number of eligible donor will decrease.^4^ A global shortage of blood supply is predicated by 2050,^5^ emphasizing the need for new sources of transfusable blood products.

As RBCs lack a nucleus and do not express HLA antigens, they are not tumorigenic and less immunogenic than most cell types. For these reasons, stem cell‐derived RBCs may be among the first to be used in clinic. Malik et al.^6^ first demonstrated the evidence that RBCs can be produced from human CD34^+^ cells ex vivo in 1998. In 2002, Douay’s group developed a procedure for large‐scale expansion of human erythroid cells from CB CD34^+^ hematopoietic stem and progenitor cells (HPSCs). This culture system resulted in a 200,000‐fold mean amplification but with inefficient enucleation of orthochromatic erythroblasts.^7^ In addition to these pioneering studies, several other groups also successfully generated RBCs from CB CD34^+^ cells,^8^ PB CD34^+^ cells,^9^ human embryonic stem cells (ES),^10^, ^11^, ^12^, ^13^ human induced pluripotent stem cells (iPS)^14^, ^15^, ^16^, ^17^ and immortalized erythroid cell lines.^18^, ^19^, ^20^, ^21^, ^22^ Although significant advances have been made during the last decade, including autologous PB CD34^+^ derived RBCs being safely tested in a human,^9^ many challenges still remain, particularly for ex vivo generation of red cells from ESs, iPSs and immortalized erythroid cell lines. Since ES/iPS possess unlimited self‐renewal ability, these cells are preferred sources to be used for ex vivo generation of red cells. However, the expansion of ES/iPS‐derived erythroid cells is limited, and the enucleation of ES/iPS‐derived erythroblasts is usually very low.^10^, ^12^, ^23^, ^24^, ^25^

Generation of human RBCs ex vivo needs to mimic a physiological process called erythropoiesis which involves commitment of HPSCs to erythroid progenitors, expansion of erythroid progenitors and terminal differentiation of erythroid progenitor CFU‐E sequentially to proerythroblasts, basophilic erythroblasts, polychromatic erythroblasts, orthochromatic erythroblasts and enucleation of orthochromatic erythroblasts. To investigate the mechanisms for the limited expansion and impaired enucleation of ES/iPS‐derived erythroid cells, in the present study we compared erythropoiesis profiles of CB CD34^+^ cells and ES CD34^+^ cells. We found that consistent with previous findings, the expansion and enucleation of erythroid cells derived from ES CD34^+^ cells were significantly lower than that derived from CB CD34^+^ cells. Mechanistically, the limited expansion of ES CD34^+^ cell‐derived erythroid cells was due to defective cell cycle of erythroid progenitors associated with decreased expression of cyclin E, CDK2 and CDK4 which promote G1 to S phase transition, and increased expression p57 which inhibits G1 to S phase progression. In exploring the cellular and molecular mechanisms for the impaired enucleation of ES‐ortho, we found the chromatin of ES CD34^+^ cell‐derived orthochromatic erythroblasts was less condensed than that of CB CD34^+^ cell‐derived orthochromatic erythroblasts. Consistent with impaired chromatin condensation, both ATAC‐seq and RNA‐seq analyses revealed that pathways involved in chromatin modification were down‐regulated. Additionally, the expression levels of molecules known to play important role in chromatin condensation or/and enucleation were significantly lower in ES‐ortho compared to that in CB‐ortho. Our findings have uncovered mechanisms for the limited expansion and impaired enucleation of ES CD34^+^ cell‐derived erythroid cells and may help to improve ex vivo RBC production from stem cells.

## 2 MATERIALS AND METHODS

### 2.1 Antibodies and other reagents for flow cytometry

Antibodies used for flow cytometry analysis were as follows: mouse monoclonal antibody against human band 3 was generated in our laboratory and labelled with FITC or APC as described previously,^26^, ^27^ PE‐conjugated CD34 (BD #555822), APC‐conjugated CD71 (BD #551374), PE‐conjugated CD235a/GPA (eBioscience #5555570) and APC‐conjugated α4 integrin (BD #130‐093‐281). Other reagents are PE‐Cy7‐conjugated annexin V (eBioscience #88‐8103‐74), Hoechst33342 (Solarbio #C0031) and 7AAD (BD #559925).

### 2.2 Cell culture and Fluorescence‐activated cell sorting

Human ES cell line H1 cells and stromal cell line OP9 cells were used in this study. Both cell lines were purchased from ATCC. H1 cells were cultured in mTeSRTM1 (Stemcell #85850) medium using Corning^®^ Matrigel^®^ hESC‐Qualified Matrix (StemCell #07181) as the surface coating matrix. The medium was changed daily. Cells were passaged using ReLeSRTM (StemCell #05872) every 4–5 days. OP9 cells were cultured in α‐MEM (Gibco #12571) with 20% FBS (BI #04002), passaged every 2–3 days. Undifferentiated H1 cells were seeded onto irradiated OP9 cells and cultured in hESCs maintenance medium.^28^, ^29^ Three days later, medium was replaced by haematopoiesis‐inducing medium.^28^, ^29^ H1 cells were co‐cultured with OP9 cells for 15 days. On Day 15, the cells were stained with PE‐conjugated CD34 and APC‐conjugated CD71 at 4℃ in the dark. The CD34^+^CD71^−^ hematopoietic stem cells were sorted using BD FACSAria fusion Cell Sorter.

### 2.3 Differentiation of ES CD34^+^ cells and CB CD34^+^ cells to erythroid cells

CD34^+^ cells were purified from CB using Ultrapure Microbeads kit according to the manufacture’s protocol (Miltenyi Biotec #130‐100‐453). Human CB samples were obtained from the third Affiliated Hospital of Zhengzhou University, China and the New York Blood Center. The enriched CB CD34^+^ cells and the sorted ES‐derived CD34^+^ cells were cultured in erythropoiesis medium for 20 days as previously described.^30^, ^31^, ^32^ Briefly, the basic medium was Iscove's Modified Dulbecco's Medium (IMDM) with 3% human AB serum, 2% human plasma, 10 μg/ml insulin, 3 IU/ml hepairn and 1% penicillin/streptomycin. The medium for Days 0–7, Days 7–11, Days 11–15 and Days 15–20 was as following respectively: 200 μg/ml Holo‐transferrin, 3 IU/ml EPO, 10 ng/ml SCF, 1 ng/ml IL‐3; 200 μg/ml Holo‐transferrin, 1 IU/ml EPO, 10 ng/ml SCF; 1 mg/ml Holo‐transferrin and 1 IU/ml EPO; 1 mg/ml Holo‐transferrin. Fresh medium was changed on Days 4, 7, 11 and 15.

### 2.4 Staining and flow cytometric analyses of erythroblasts differentiation, apoptosis and cell cycle

Flow cytometric analyses for examining the in vitro terminal erythroid differentiation were performed as previously described.^26^, ^31^ Annexin V kit (eBioscience #88‐8103‐74) was used for apoptosis detection according to the manufacturer’s protocol. EdU flow cytometry assay kit (Invitrogen No.MAN0009883) was used for cell cycle detection according to the previous paper.^27^

### 2.5 Amnis ImageStream flow cytometry

To examine cell and chromatin condensation, 0.5 × 10^6^ erythroblasts were stained with 5 μM Hoechst 33342 at 37℃ for 30 min. The cells were stained with PE‐conjugated GPA, FITC‐conjugated Band3 and APC‐conjugated α4 integrin. Cells were washed with 1 ml PBS/2%FBS/2 mM EDTA buffer by centrifugation at 300 *g* for 5 min, at 4℃. The cells were then resuspended in 50 μl PBS/2% FBS/2 mM EDTA buffer. Cells were analysed within 1 h using Amnis ImageStream analysis. Data were collected on an Amnis ImageStream Mark II instrument using a 60× objective. IDEAS software was used to analyse the data.

### 2.6 Erythroid colony assays, cytospin and May‐Grunwald‐Giemsa staining

Cells were diluted at a density of 200 cells in 1 ml of MethoCult H4330 (Stemcell #04330) medium for CFU‐E colony assay and MethoCult H4434 (Stemcell #04434) for BFU‐E colony assay, and incubated at 37℃ in a humidified atmosphere incubator with 5% CO_2_ in air. CFU‐E colonies were counted under an inverted microscope on Day 7 after plating. BFU‐E colonies were counted on Day 14 after plating. Cytospin and May‐Grunwald‐Giemsa staining were performed as described in our previous studies.^26^ Images were taken using Leica DM2000 inverted microscopy at 40× or 100× magnification. Cells were analysed with ImageJ software.

### 2.7 RNA‐seq

RNA was extracted from ES‐derived Day 17 erythroblasts which are orthochromatic erythroblasts using QIAGEN RNA isolation kit (QIAGEN#74104) according to the manufacturer's instructions. The RNA integrity number of each sample was >9. Approximately 100 ng of total RNA was used for construction of cDNA library by Illumina TruSeq kit (Illumina #RS‐121‐2001, #RS‐121‐2002). Sequencing was performed on Illumina HiSeq 4000 with 150 bp paired‐end. RNA‐Seq data of CB‐derived erythroblasts were downloaded from our previous data.^33^ Raw bulk RNA‐seq data of ES‐ortho were filtered by fastp to remove low quality reads. Reads were mapped to UCSC hg19 by STAR^34^ and quantified by FeatureCounts.^35^ Gene expression was analysed using DESeq2.^36^ Principal component analysis (PCA) was performed on log‐transformed normalized counts for expressed genes. Differentially expressed genes were identified as fold change ≥4 and adjusted *p*‐value ≤0.05. GO (Gene ontology) enrichment analysis were performed by Metascape.^37^ GO term was considered as significant with *q*‐value <0.001. RNA‐seq data are available at GEO under accession number GSE179778. Time series analysis was applied by R package TCseq.

### 2.8 ATAC‐seq

Nuclei were extracted from 50,000 cells of FACS‐sorted CB‐derived orthochromatic erythroblasts and ES‐derived D17 orthochromatic erythroblasts. The transposition reaction was performed with Tn5 transposase, and the transposed DNA was purified and amplified using a Zymo DNA clean and concentrator‐5 kit (Zymo#D4014) to generate sequencing libraries. Amplified DNA fragments from 150 to 1300 bp range were purified and sequenced by Illumina HiSeq 4000 with 150 bp paired‐end. Raw reads were filtered by fastp and then mapped to UCSC hg19 genome using Bowtie2.^38^ Reads mapping to ENCODE blacklist regions and the mitochondrial genome were removed. Peaks were called using MACS2^39^ with parameters ‐‐nomodel ‐‐shift −100 ‐‐extsize 200. Differentially accessible peaks (DAPs) were identified using the DiffBind^40^ as fold change ≥2 and adjusted *p*‐value ≤0.01. Peaks were annotated to the nearest gene transcription start site (TSS) using ChIPseeker.^41^ Association of differentially expressed genes and DAPs were calculated by Binding and Expression Target Analysis (BETA).^42^ GO enrichment of DAP was performed by GREAT.^43^ ATAC‐seq data are available at GEO under accession number GSE179778.

### 2.9 Statistical analysis

FlowJo software was used to analyse FCS data. ImageJ was used to analyse band signal intensities. GraphPad Prism software was used for statistical analysis. All data were reported as mean ± SEM. Differences among two groups were calculated by unpaired Student’s *t*‐test.

## 3 RESULTS

### 3.1 Generation of CD34^+^ HPSCs from ES cells

To generate erythroid cells from ES cell‐derived stem cells, we first need to generate CD34^+^ HPSCs from ES cells. For this, we employed the well‐established co‐culture system by co‐culturing the human ES cell line H1 cells with the OP9 stromal cells^28^, ^29^ (Figure S1A). Figure S1B shows that on Day 15 of the H1‐OP9 co‐culture, there were ~21% of CD34^+^CD71^−^ cells. The CD34^+^CD71^−^ (will refer to as ES‐CD34^+^ cells thereafter) were sorted by FACS and used for further erythroid differentiation.

### 3.2 Defective cell cycle of the ES CD34^+^ cell‐derived erythroid cells

**FIGURE 1:**
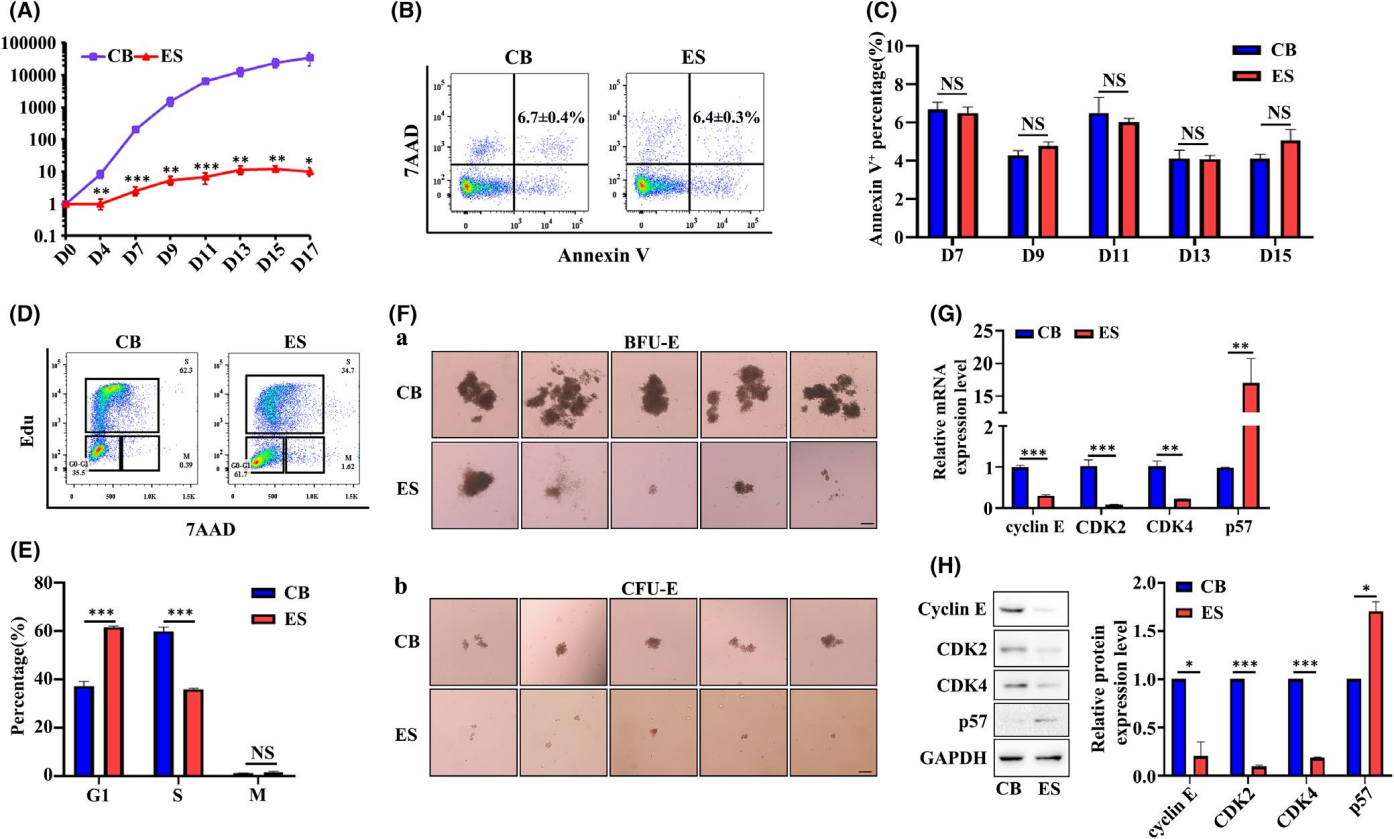
Proliferation potential of erythroid cells derived from CB CD34^+^ or ES CD34^+^ cells. (A) Growth curve of erythroid cells derived CB CD34^+^ or and ES CD34^+^ cells in 3 phase erythroid culture medium (*N* = 6). (B) Representative flow cytometry profiles of apoptosis as assessed by Annexin V and 7AAD staining of Day 7 erythroid cells. (C) Quantitative analyses of apoptosis. *N* = 3. (D) Representative flow cytometry profiles of cell cycle as assessed by Edu and 7AAD staining of Day 7 erythroid cells. (E) Quantitative analyses of cell cycle. *N* = 3. (F) Colony forming ability of Day 4 erythroid cells derived from CB CD34^+^ or ES CD34^+^ cells in BFU‐E colony medium or CFU‐E colony medium. Scale bar, 200 μm. (G) mRNA levels (normalized to actin) of cyclin E, CDK2, CDK4 and p57, as assessed by qRT‐PCR. (H) Representative Western blot analysis of cyclin E, CDK2, CDK4 and p57 (left panel) and quantitative analysis of relative protein expression levels from three independent experiments (right panel). *p < 0.05, **p < 0.01, ***p < 0.001

It has been reported previously that CD34^+^ cells from CB, PB or BM can expand 1 × 10^5^–2 × 10^6^ folds when differentiated to erythroid cells.^8^, ^32^ In marked contrast, ES or iPS‐derived HSPCs can only expand 10–50 times.^44^, ^45^ To investigate the reasons for the limited expansion capacity of ES/iPS‐derived erythroid cells, we compared the in vitro erythroid differentiation profiles of ES CD34^+^ cells and CB CD34^+^ cells using the three‐phase erythroid differentiation protocol.^30^, ^31^ We first monitored cell growth. Figure 1A shows that consistent with previous studies,^44^, ^45^ ES CD34^+^ cells yielded 3500‐fold less erythroid cells than CB CD34^+^ cells under our experimental conditions (10.06 ± 1.8 fold vs. 35,346 ± 6866 fold, *p* < 0.05). The impaired cell growth could be due to increased cell death, or decreased cell proliferation or both. To examine the cause for the impaired cell growth, we first measured apoptosis. The representative profiles of dual staining of 7AAD and Annexin V on Day 7 erythroid cells are shown in Figure 1B. Quantitative analyses revealed no significant differences in the percentage of Annexin V^+^ cells between the two groups (Figure 1C). We next assessed cell cycle. The representative profiles of cell cycle analysis on D7 erythroid cells are shown in Figure 1D. Quantitative analyses revealed that ~38% and ~60% of CB CD34^+^ cell‐derived erythroid cells were in G1 phase and S phase respectively (Figure 1E). In contrast, ~60% and ~37% of ES CD34^+^ cell‐derived erythroid cells were in G1 phase and S phase respectively (Figure 1E). These findings indicate that the impaired cell growth of ES CD34^+^ cell‐derived erythroid cells is associated with defective proliferation but not with apoptosis. The slope of growth curve suggests that defective proliferation is manifested from erythroid progenitor stage. To test this, we performed erythroid colony assay. As expected, Figure 1F shows that the sizes of ES CD34^+^ cell‐derived BFU‐E and CFU‐E colonies were much smaller than that from CB CD34^+^ cell‐derived BFU‐E and CFU‐E colonies. To explore the reasons for the defective G1‐S transition, we examined the expression of key genes involved in G1‐S transition by both real‐time PCR and Western blot. We found that cyclin E, CDK2 and CDK4, which promote G1 to S phase transition, were significantly lower in Day 7 ES‐erythroid cells compared to that in Day 7 CB‐erythroid cells. In contrast, p57, which inhibits G1 to S phase progression, was significantly higher in Day 7 ES‐erythroid cells compared to that in Day 7 CB‐erythroid cells (Figure 1G,H).

### 3.3 Accelerated terminal erythroid differentiation of ES CD34^+^ cells

**FIGURE 2:**
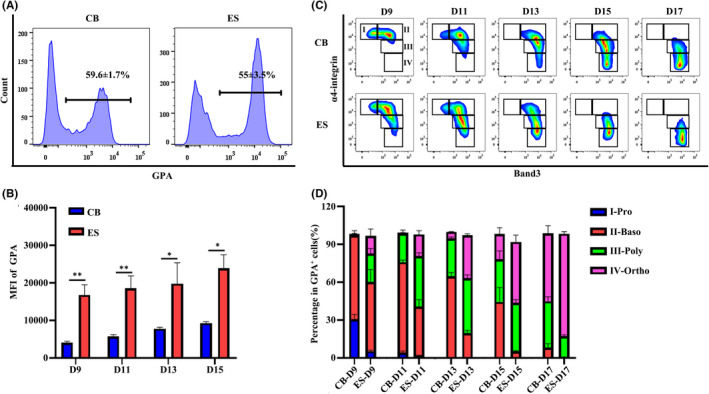
Terminal erythroid differentiation of CB CD34^+^ or ES CD34^+^ cells. (A) Flow cytometry analysis showing the percentage of GPA^+^ cells of Day 9 erythroid cells. (B) Quantitative analysis showing the mean fluorescent intensity (MFI) of GPA. (C) Representative flow cytometry analysis of band3 and α4‐integrin expression on GPA^+^ erythroblasts. (D) Quantitative analyses of erythroblasts at distinct developmental stages based on the expression of band3 and α4‐integrin shown in Figure 2C. *N* = 3. *p < 0.05, **p < 0.01

We next monitored terminal erythroid differentiation by flow cytometry using glycophorin A (GPA), band 3 and α4 integrin as surface markers. Figure 2A shows representative flow cytometry analyses of GPA expression on erythroblasts cultured for 9 days. Quantitative analyses reveal that ES CD34^+^ cell‐derived erythroblasts expressed higher levels of GPA comparing to that derived from CB CD34^+^ cells (Figure 2B). Terminal erythroid differentiation was further assessed using band 3 and α4 integrin as surface markers.^26^, ^27^, ^30^, ^31^ Representative plots of band 3 versus α4 integrin of the GPA^+^ cells were shown in Figure 2C. As described previously,^26^, ^27^, ^30^, ^31^ based on the expression pattern of band 3 and α4 integrin, erythroblasts can be separated into 4 distinct populations: proerythroblasts (I), basophilic erythroblasts (II), polychromatic erythroblasts (III) and orthochromatic erythroblasts (IV). Quantitative analyses of erythroblasts at distinct developmental stage were shown in Figure 2D. It reveals that on Day 9 of culture, while CB CD34^+^ cell‐derived GPA^+^ erythroblasts were mostly at proerythroblast and basophilic erythroblast stage, some of ES CD34^+^ cell‐derived erythroblasts already differentiated to polychromatic erythroblasts. On Day 11 and Day 13 of culture, while the majority of CB CD34^+^ cell‐derived GPA^+^ erythroblasts were still at basophilic erythroblasts, ES CD34^+^ cell‐derived erythroblasts already contained polychromatic erythroblasts and orthochromatic erythroblasts. Similarly, on Days 15 and 17, ES CD34^+^ cell‐derived erythroblasts contained more polychromatic and orthochromatic erythroblasts than CB CD34^+^ cell‐derived erythroblasts. Together, these findings demonstrate accelerated terminal erythroid differentiation of ES CD34^+^ cells comparing to that of CB CD34^+^ cells.

### 3.4 Impaired enucleation and chromatin condensation of ES CD34^+^ cell‐derived erythroblasts

**FIGURE 3:**
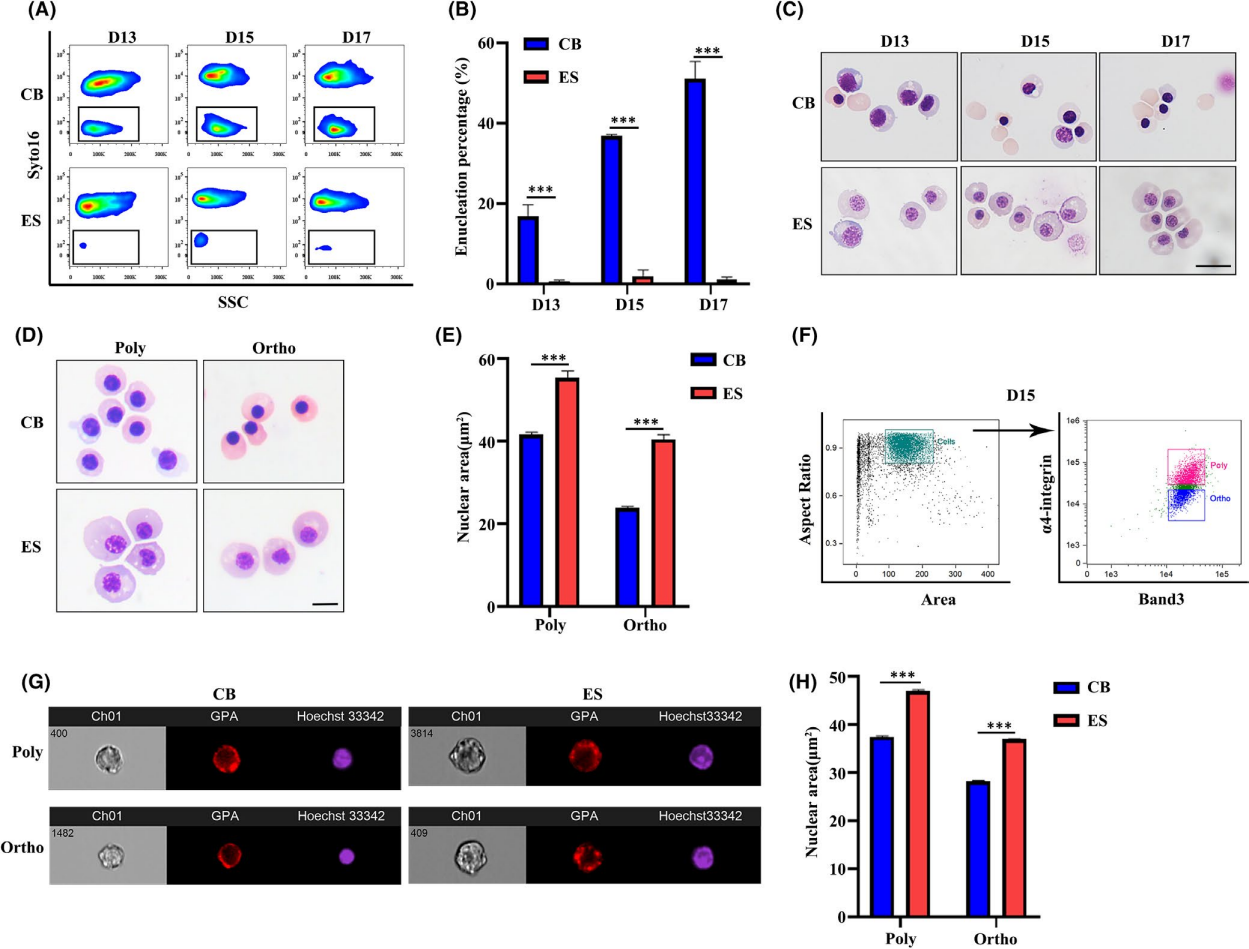
Impaired enucleation and chromatin condensation of orthochromatic erythroblasts derived from ES CD34^+^ cells. (A) Representative flow cytometry analyses of enucleation as assessed by Syto16 staining. (B) Quantitative analyses of enucleation. (C) Representative cytospin images of erythroblasts. Scale bar, 20 μm. (D) Representative cytospin images of polychromatic and orthochromatic erythroblasts. Scale bar, 10 μm. (E) Quantitative analyses of nuclear area (μm^2^) of poly and ortho erythroblasts by ImageJ. (F) Representative ImageStream analysis of band 3 and α4‐integrin expression on Day 15 of GPA^+^ erythroblasts. (G) Representative ImageStream images of poly and ortho erythroblasts. (H) Quantitative analyses of nuclear area (μm^2^) of poly and ortho erythroblasts by ImageStream. *N* = 3. ***p < 0.001

Enucleation is the last step of terminal erythroid differentiation. Next, we assessed enucleation by flow cytometry.^46^, ^47^ Representative enucleation profiles are shown in Figure 3A. Quantitative analyses (Figure 3B) show that under our erythroid culture conditions, CB CD34^+^ cell‐derived erythroblasts started to enucleate on Day 13, and the percentage of enucleated cells reached to ~50% on Day 17 of culture. In marked contrast, very few ES CD34^+^ cell‐derived erythroblasts were able to enucleate. The inability of ES CD34^+^ cell‐derived erythroblasts to enucleate was also demonstrated by cytospin (Figure 3C). In preparation for enucleation, late‐stage erythroblasts must condense their nuclei. To examine whether the inability of ES CD34^+^ cell‐derived erythroblasts to enucleate is associated with chromatin condensation, we next examined the chromatin condensation status of polychromatic and orthochromatic erythroblasts. Representative cytospin images of polychromatic and orthochromatic erythroblasts are shown in Figure 3D which shows clearly that the chromatin of ES CD34^+^ cell‐derived polychromatic (ES‐poly) and orthochromatic erythroblasts (ES‐ortho) was less condensed than the corresponding polychromatic erythroblasts or orthochromatic erythroblasts derived from CB CD34^+^ cells. Quantification of nuclear area using Image J revealed that the nuclear areas of ES‐poly and ES‐ortho were significantly larger than that of CB‐poly and CB‐ortho (Figure 3E). We also quantified the size of the cell, the cytoplasm and nuclear/cytoplasmic ratio. Interestingly, ES‐poly and ES‐ortho were much larger than CB‐poly and CB‐ortho, (Figure S3A), and they also have larger cytoplasm (Figure S3B) and lower nuclear/cytoplasmic ratio (Figure S3C), reflecting characteristics of embryonic erythropoiesis.^10^ We further assessed chromatin condensation using Image Flow Cytometry (ImageStream) analyses. Similar to the conventional flow cytometry analysis, polychromatic and orthochromatic erythroblasts were gated based on the expression pattern of band 3 and α4 integrin (Figure 3F). Representative ImageStream images are shown in Figure 3G. More images used for the quantification were shown in Figure S2. Quantitative analyses show that consistent with results obtained from cytospin analyses, ES‐poly and ES‐ortho had larger nuclear size than CB‐poly and CB‐ortho (Figure 3H). These results demonstrate impaired enucleation and chromatin condensation of ES CD34^+^ cell‐derived erythroblasts.

### 3.5 Down‐regulation of pathways involved in chromatin modification and autophagy in ES‐ortho

**FIGURE 4:**
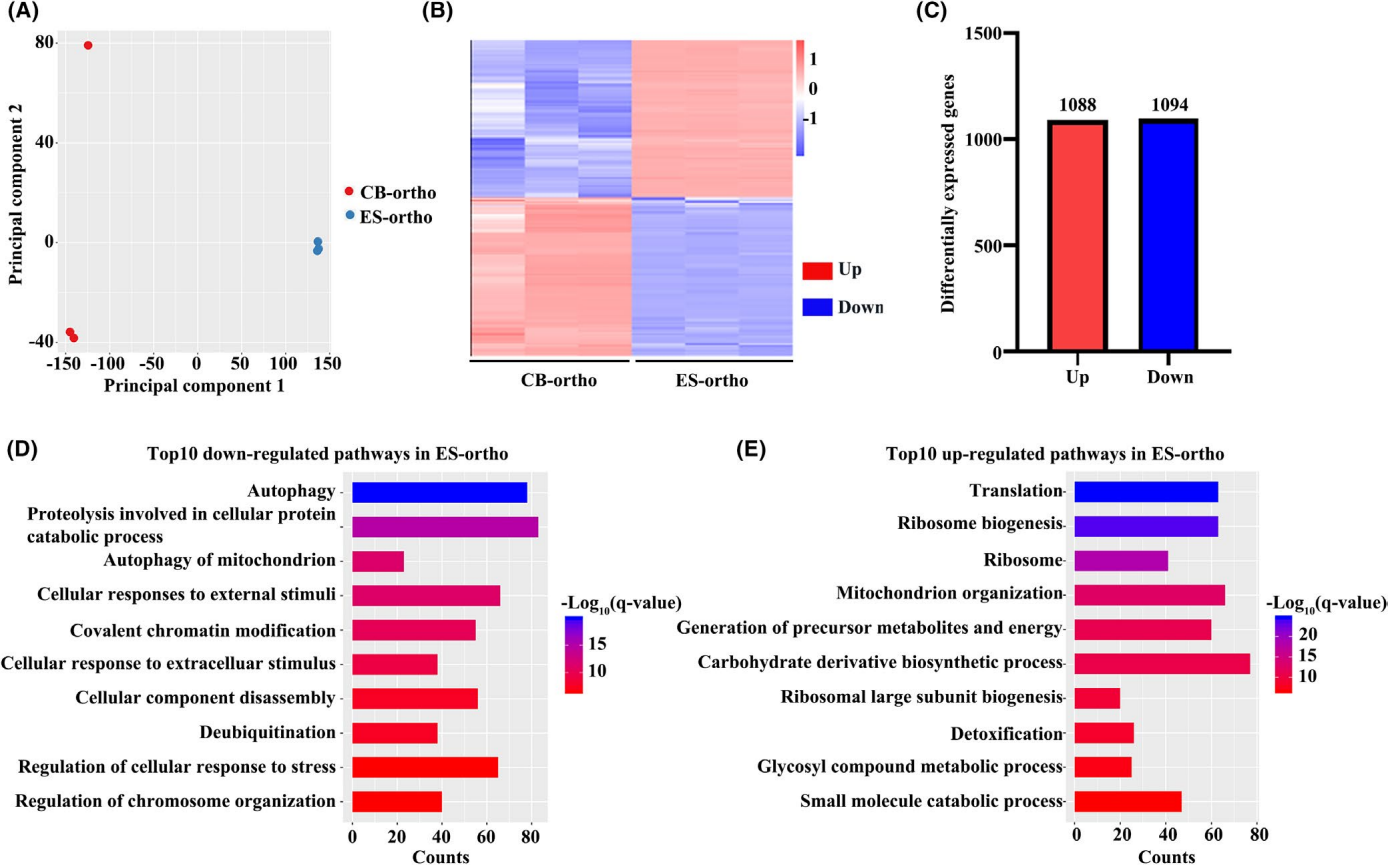
RNA‐seq analyses of CB and ES orthochromatic erythroblasts. (A) Principal component analyses. (B) Heat map of differentially expressed genes. (C) Bar plot of differentially expressed gene numbers. (D) Top 10 down‐regulated pathways in ES‐ortho. (E) Top 10 up‐regulated pathways in ES‐ortho. The colour represents log‐transformed adjusted *q*‐value; the width indicates the number of differentially expressed genes in the category

To investigate the molecular basis for the impaired chromatin condensation and enucleation, we performed RNA‐seq analyses on ES‐ortho and compared them to CB‐ortho RNA‐seq we generated previously.^33^, ^48^ Principal component analyses (PCA) of the RNA‐seq data demonstrated a high degree of separation between ES‐ortho and CB‐ortho samples (Figure 4A). A heat map of differentially expressed genes (DEGs) between ES‐ortho and CB‐ortho is shown in Figure 4B. Figure 4C shows that 2182 genes were differentially expressed, with 1088 up‐regulated and 1094 down‐regulated. DEGs are listed in Table S1. GO analyses of the DEGs revealed that consistent with impaired chromatin condensation, pathways involved in chromatin modification were among the top down‐regulated pathways in ES‐ortho compared to CB‐ortho (Figure 4D). DEGs involved in chromatin condensation are listed in Table S2. Additionally, pathways involved in autophagy were also down‐regulated in ES‐ortho (Figure 4D). DEGs involved in autophagy are listed in Table S3. In contrast, pathways involved in translation and ribosome biogenesis were up‐regulated in ES‐ortho (Figure 4E). To get a more comprehensive view of the transcriptional status of ES‐ortho, we also compared the ES‐ortho with all terminal stages of CB‐derived erythroblasts. Differentially expressed genes from adjacent pairwise comparison (CB‐proerythroblasts, CB‐early‐stage basophilic erythroblasts, CB‐late‐stage basophilic erythroblasts, CB‐polychromatic erythroblasts, CB‐orthochromatic erythroblasts and ES‐orthochromatic erythroblasts) were analysed, and 3863 differentially expressed genes were identified. Those DEGs were clustered into six groups by time series analysis TCseq. Their expression pattern and GO enrichment were shown in Figure S4 and Table S6. Among these six clusters, genes in cluster 1/3/4 had divergent expression in ES‐ortho comparing to the global tendency of terminal CB‐derived erythroblasts, which were likely to be the truly differentially expressed genes between ES‐ and CB‐derived erythroblasts. 85.6% of DEGs between ES‐ortho and CB‐ortho belonged to cluster 1/3/4. We also found that GO enrichment of cluster 3 and cluster 4 were consistent with GO enrichment of up‐regulated and down‐regulated genes in ES‐ortho comparing to CB‐ortho. Overall, the cluster analysis verified that ES‐derived orthochromatic erythroblasts were down‐regulated in chromatin modification and autophagy, and up‐regulated ribosome biogenesis.

### 3.6 Differential chromatin accessibility between ES‐Ortho and CB‐Ortho

**FIGURE 5:**
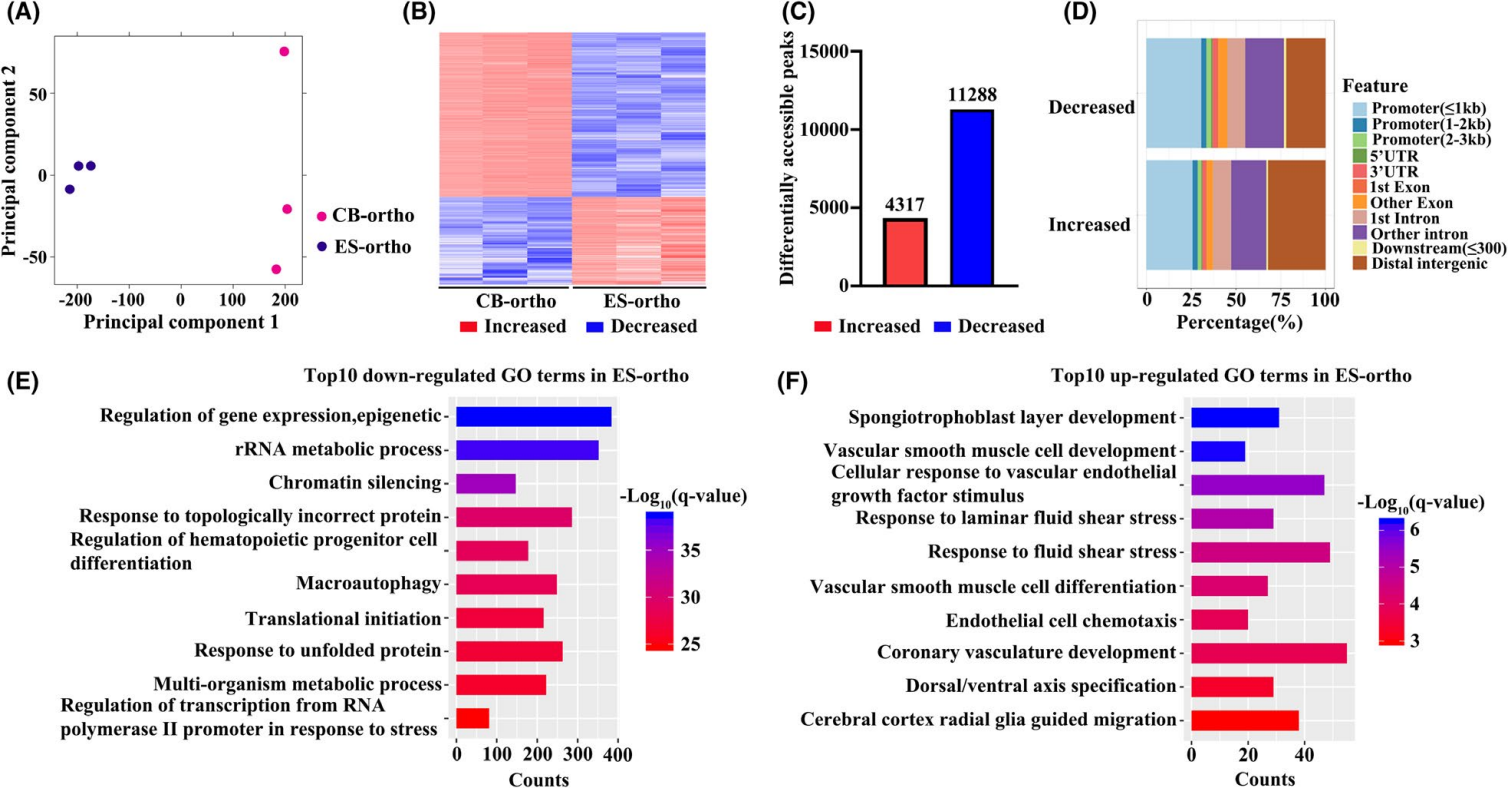
ATAC‐seq analyses of CB‐ortho and ES‐ortho. (A) Principal component analyses. (B) Heat map of differential chromatin accessibility. (C) Numbers of increased and decreased accessible peaks. (D) The distribution of peaks relative to gene features for ES‐ortho increased and decreased accessible peaks. (E) Enriched GO terms of decreased accessible peaks in ES‐ortho. (F) Enriched GO terms of increased accessible peaks in ES‐ortho. The colour represents adjusted binomial *q*‐value, and the bar width indicates the number of differentially accessible peaks in the category

Next, we performed ATAC‐seq to compare the differences in chromatin accessibility between ES‐ortho and CB‐ortho. PCA revealed a high degree of separation between ES‐ortho and CB‐ortho (Figure 5A). Peaks were assigned to the nearest gene based on annotated TSS by ChIPseeker. A heat map of DAPs was shown in Figure 5B. Figure 5C showed that 4317 peaks had increased accessibility and 11,288 peaks had decreased accessibility in ES‐ortho compared to CB‐ortho. DAPs were listed in Table S4. We also examined the distribution of DAPs relative to gene features and found that DAPs that were decreased in ES‐ortho had a higher presence in the promoter regions and a lower presence in distal intergenic regions compared to DAPs that were increased in ES‐ortho (Figure 5D). GO analyses of DAPs revealed that similar to RNA‐seq, pathways involved in macro‐autophagy and chromatin modification were among the top down‐regulated pathways in ES‐ortho (Figure 5E). In contrast, pathways involved in cellular response to vascular endothelial growth factor stimulus and response to endothelial cell chemotaxis were up‐regulated in ES‐ortho (Figure 5F).

### 3.7 Association between gene expression and chromatin accessibility

**FIGURE 6:**
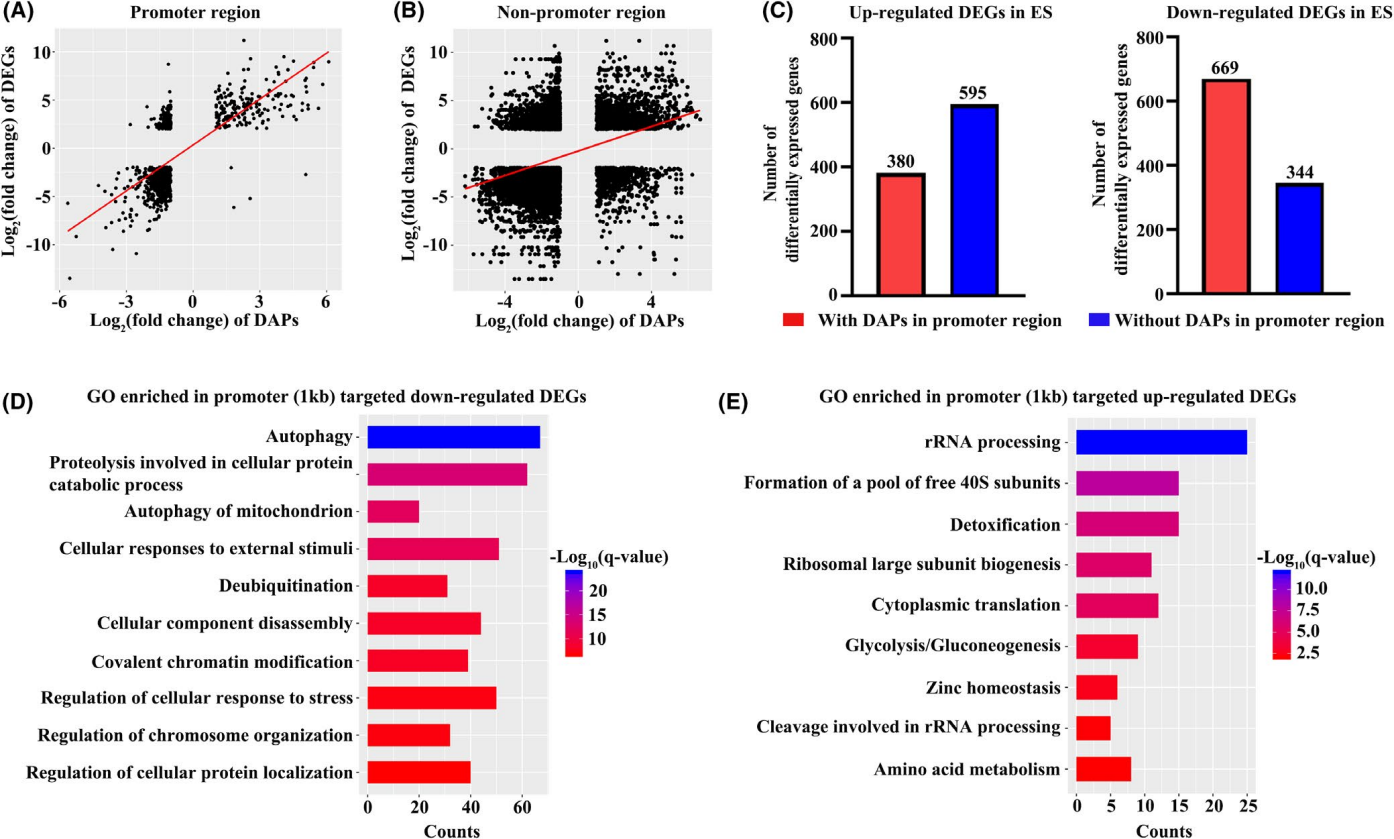
Correlation of open chromatin and gene expression. (A) The log_2_(fold change) of differentially accessible peaks (DAPs) plotted against the log_2_(fold change) of differentially expressed genes (DEGs) with DAPs at promoter regions. (B) The log_2_(fold change) of DAPs plotted against the log_2_(fold change) of DEGs with DAPs at non‐promoter regions. (C) Up‐regulated and down‐regulated differentially expressed gene numbers with or without DAPs at promoter regions. (D) Bar plot of enriched GO terms of down‐regulated DEGs with DAPs at promoter regions. (E) Bar plot of enriched GO terms of up‐regulated DEGs with DAPs at promoter regions. The colour represents adjusted binomial *q*‐value, and the bar width indicates the number of differentially accessible peaks in the category

Chromatin accessibility at gene promoters is expected to be associated with gene expression activation or repression. To assess the association between chromatin accessibility and gene expression in ES‐ortho and CB‐ortho, we used BETA^42^ to integrate ATAC‐seq DAPs with differential gene expression data to infer potential targets of DAPs. We found that ~91% of DEGs (1988 in 2182) were potentially regulated by DAPs. The predicted association between DAPs and DEGs was listed in Table S5. One DEG could be associated with several DAPs at its promoter region or non‐promoter region. We further checked the relationship between DEGs and DAPs within promoter region, and found that ~48% of DEGs were associated with DAPs at promoter regions (distance to TSS ≤ 1 kb). Correlation of fold changes between DEGs and DAPs was calculated. The results showed stronger correlation (coefficient: 0.70, Figure 6A) in promoter regions than non‐promoter regions (coefficient: 0.32) (Figure 6B). Numbers of DEGs with or without DAPs at promoter regions were displayed in bar plot. ES‐ortho down‐regulated DEGs showed higher presence of DAPs at promoter region than up‐regulated DEGs with *p*‐value ≤0.0001 (Figure 6C). Next, we analysed GO enrichment of DEGs with DAPs at promoter region. Similar to RNA‐seq and ATAC‐seq analyses, ES‐ortho down‐regulated DEGs with DAPs at promoter regions were enriched in autophagy and chromatin modification (Figure 6D), while ES‐ortho up‐regulated DEGs with DAPs at promoter regions were enriched in rRNA processing and formation of free 40S subunits (Figure 6E).

### 3.8 Expression levels of molecules known to be involved in chromatin condensation or/and enucleation are decreased in ES‐ortho due to decreased chromatin accessibility at their promoter regions

**FIGURE 7:**
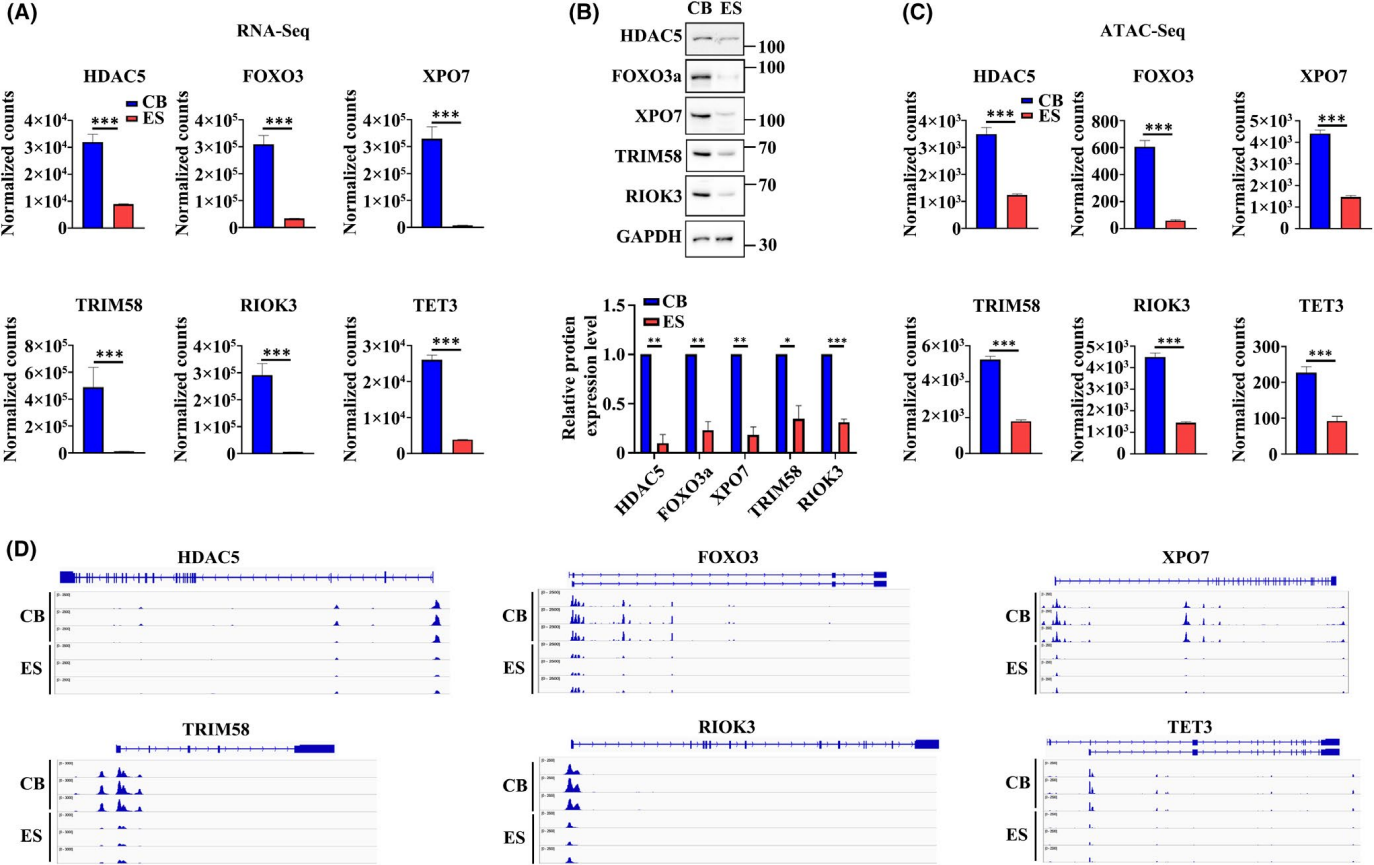
Expression of molecules known to be involved in enucleation. (A) The normalized count value of differentially expressed genes (DEGs) in ES‐ortho and CB‐ortho from RNA‐seq as indicated. (B) Western blot analysis showing protein level differences between ES‐ortho and CB‐ortho. Quantitative analysis of protein expression levels from three independent experiments is shown (bottom panel). (C) The normalized count value of differentially accessible peaks (DAPs) in ES‐ortho and CB‐ortho from ATAC‐seq as indicated. (D) The reads distribution of differentially accessible peaks at promoter regions of genes as indicated. *p < 0.05, **p < 0.01, ***p < 0.001

In exploring the molecular mechanisms for the mammalian erythroblast enucleation, previous studies have identified molecules that play important roles in chromatin condensation or/and enucleation. These include histone deacetylase family members HDAC2,^49^ HDAC6,^50^ HDAC5,^51^ DNA demethylase TET3,^31^ transcription factor FOXO3,^52^ the nuclear export protein exportin 7(XPO7),^53^ an E3 ubiquitin ligase TRIM58,^54^ Rac GTPases (Rac1 and Rac2) and their effector mDia2,^55^ and the atypical protein kinase RIOK3.^56^ In addition to global pathway analyses, we selectively examined the expression levels of these genes and their chromatin accessibility. As shown in Figure 7A, the expression levels of some of the above‐mentioned genes such as HDAC5, FOXO3, XPO7, TRIM58, RIOK3 and TET3 were significantly lower in ES‐ortho compared to CB‐ortho. The decreased expression levels of these molecules were confirmed at protein level (Figure 7B). Notably, the decreased expression of these genes was accompanied by the decreased chromatin accessibility at their promoter regions (Figure 7C). Representative chromatin accessibility profiles of these genes are shown in Figure 7D.

Additionally, while expression levels of haemoglobin genes (HBA, HBB and HBG) were much lower in ES‐ortho, the expression levels of haemoglobin genes HBE1 and HBZ, which reflect characteristics of embryonic erythropoiesis, were much higher in ES‐ortho compared to CB‐ortho (Figure S5A). The gene expression level was positively associated with chromatin accessibility at their distinct promoter region (Figure S5B). Representative chromatin accessibility profiles of haemoglobin genes are shown in Figure S5C.

## 4 DISCUSSION

Ex vivo generation of RBCs from stem cells has the potential to circumvent the shortfalls in global demand for blood transfusion. Several cell sources have been used to generate RBCs. These include CD34^+^ cells from CB,^7^, ^8^ adult bone marrow,^8^ PB,^9^ ES cells,^10^, ^11^, ^12^, ^13^ iPS cells^14^, ^15^, ^16^, ^17^ and immortalized erythroid cell lines.^18^, ^19^, ^20^, ^21^, ^22^ As ES and iPS cells possess self‐renewal ability, they can provide unlimited cell sources for such application. However, comparing to erythroid cells derived from CB/PB/BM CD34^+^ cells, the expansion and enucleation of ES/iPS‐derived erythroid cells have been shown to be very low.^10^, ^12^, ^23^, ^24^, ^25^ To understand the mechanisms for these defects, in the present study we performed detailed cellular and molecular characterizations of erythroid cells derived from ES CD34^+^ cells, using erythroid cells derived from CB CD34^+^ cells as controls. We documented that the limited expansion of ES CD34^+^ cell‐derived erythroid cells is associated with impaired proliferation of erythroid progenitors due to cell cycle defects. We further documented that defective enucleation of ES‐ortho is associated with down‐regulation of pathways involved in chromatin modification and decreased expression of multiple genes known to be involved in enucleation. Our findings have implications in helping development of strategies to improve *ex vivo* RBC production.

In exploring the mechanisms for the limited expansion of ES CD34^+^‐derived erythroid cells, we found that the limited expansion is not due to increased apoptosis but rather associated with limited proliferation of erythroid cells due to defective cell cycle, starting from erythroid progenitor stage. We further found that the expression levels of genes promoting transition from G1 to S phase during cell cycle such as cyclin E, CDK2 and CDK4 were significantly lower, while p57 which inhibits G1‐S transition was significantly higher in Day 7 ES‐erythroid cells comparing to CB‐erythroid cells. Others and we have shown that mouse CFU‐E and early‐stage erythroblasts Pro and Baso are highly proliferative with 60–90% cells in S phase.^57^, ^58^, ^59^ Here, we show that ~60% of human CB CD34^+^ cell‐derived Day 7 erythroid cells are in S phase. In contrast, less than ~40% of human ES CD34^+^ cell‐derived Day 7 erythroid cells are in S phase. Our findings are consistent with previous report that the expression levels of cell cycle‐related genes are significantly lower in ES‐derived early‐stage erythroid cells compared to that in CB‐derived cells.^44^ These findings suggest that strategies to enhance proliferation of erythroid progenitors and/or to promote cell cycle progression can be used to improve expansion capacity of ES‐derived erythroid cells.

Chromatin condensation plays a critical role in enucleation.^60^, ^61^ Previous studies have shown that impaired chromatin condensation contributes to defective enucleation.^49^, ^62^ Our finding that the chromatin of ES‐ortho was much less condensed comparing to that of CB‐ortho strongly suggests that impaired chromatin condensation of ES‐ortho contributes to its defective enucleation. Consistent with the observed impairment in chromatin condensation, both RNA‐seq and ATAC‐seq analyses revealed that pathways involved in chromatin modification are down‐regulated in ES‐ortho compared to CB‐ortho.

Previous studies have documented the important roles of several molecules in enucleation. These include XPO7,^53^ TRIM58,^54^ RIOK3,^56^ FOXO3,^52^ TET3^31^ and HDAC5.^51^ In the present study, we found that the expression levels of these molecules were significantly lower in ES‐ortho compared to CB‐ortho due to the decreased chromatin accessibility at their promoter regions. These findings demonstrate the correlation between gene expression and chromatin accessibility at the promoter region, and suggest that strategies to activate the expression of these genes in ES‐derived erythroid cells may help to improve enucleation.

Autophagy plays an important role during the final stage of the erythroid differentiation through clearance of unnecessary organelles such as ribosome and mitochondrial(mitophagy) allowing the correct formation of mature RBCs.^63^ Notably, another top down‐regulated pathway in ES‐ortho comparing to CB‐ortho was autophagy which was accompanied by the up‐regulation of pathways involved in ribosome biogenesis and mitochondrion organization. It will be interesting to investigate whether autophagy pathway play a role in enucleation.

## CONFLICT OF INTEREST

The authors declare no competing financial interests.

## AUTHOR CONTRIBUTIONS

**Shihui Wang:** Conceptualization (lead); data curation (lead); formal analysis (lead); investigation (lead); methodology (lead); resources (lead); software (lead); supervision (lead); validation (lead); visualization (lead); writing – original draft (lead). **Huizhi Zhao:** Conceptualization (lead); data curation (lead); formal analysis (lead); software (lead); validation (lead); visualization (lead); writing – original draft (equal). **Huan Zhan:** Investigation (equal); methodology (equal); validation (equal). **Chengjie Gao:** Investigation (equal); methodology (equal); validation (equal). **Xinhua Guo:** Investigation (equal); methodology (equal); resources (equal); supervision (equal). **Lixiang Chen:** Project administration (equal); supervision (equal). **Cheryl Lobo:** Project administration (equal); supervision (equal). **Karina Yazdanbakhsh:** Project administration (equal); supervision (equal). **Shijie Zhang:** Project administration (equal); supervision (equal); writing – review and editing (equal). **Xiuli An:** Funding acquisition (lead); project administration (lead); supervision (lead); writing – review and editing (lead).

## Supporting information

Fig S1

Fig S2

Fig S3

Fig S4

Fig S5

Table S1

Table S2

Table S3

Table S4

Table S5

Table S6
