# Supplementary figures and images for: Analyses of erythropoiesis from embryonic stem cell‐CD34+ and cord blood‐CD34+ cells reveal mechanisms for defective expansion and enucleation of embryomic stem cell‐erythroid cells
Source: J Cell Mol Med. 2022 Mar 5;26(8):2404–16. doi: 10.1111/jcmm.17263 (PMC8995447; doi:10.1111/jcmm.17263)

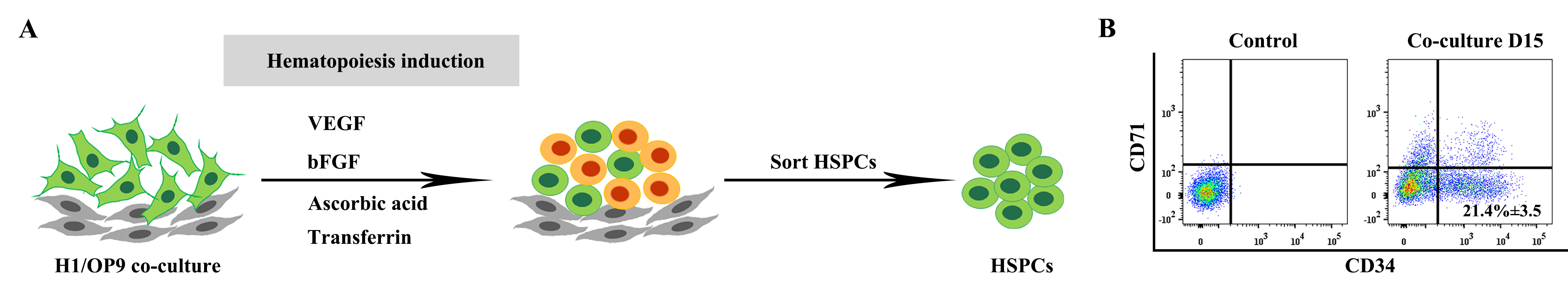

Supplement: Supplementary file 1 — Fig S1 [file JCMM-26-2404-s003.png]

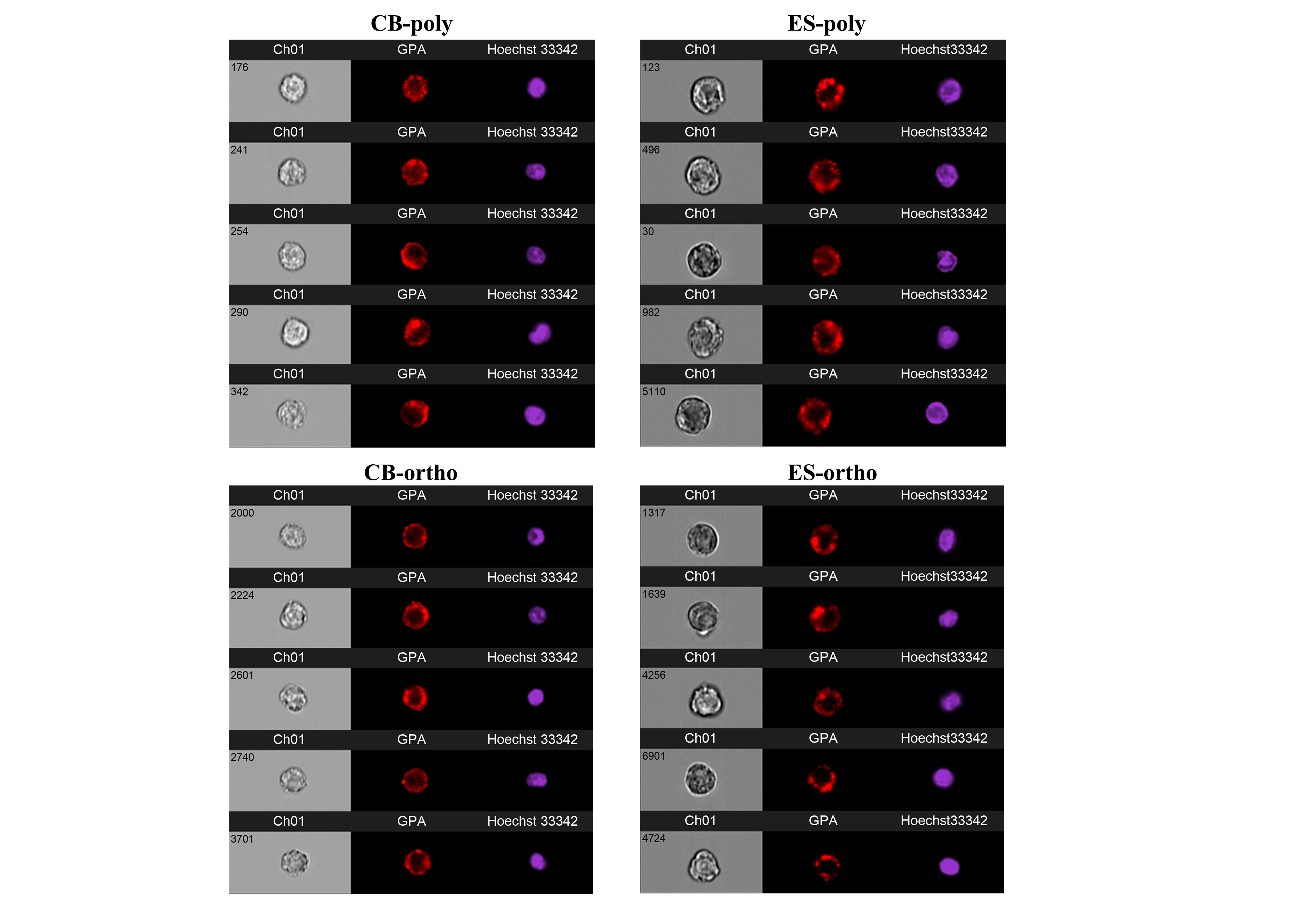

Supplement: Supplementary file 2 — Fig S2 [file JCMM-26-2404-s008.png]

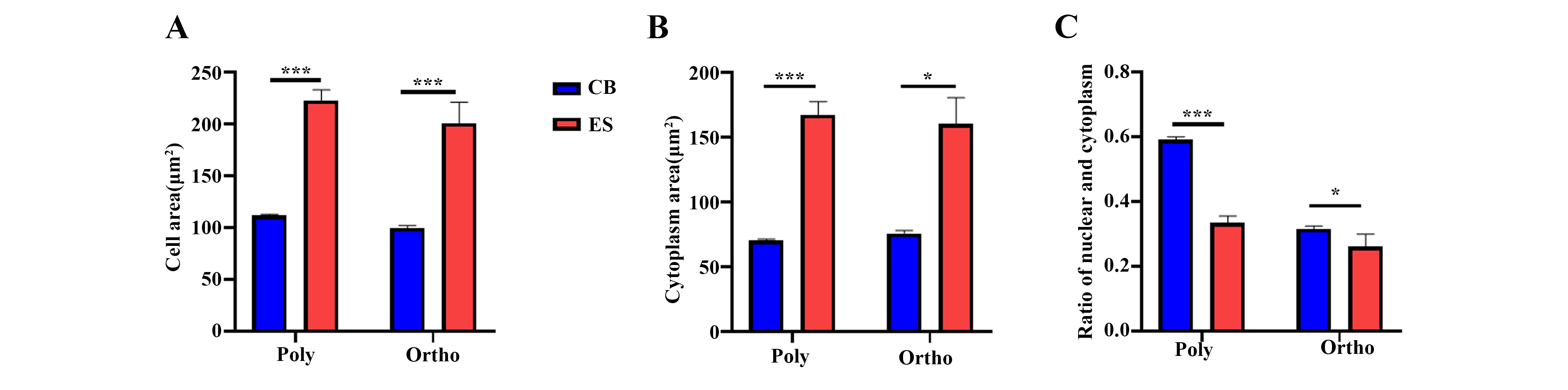

Supplement: Supplementary file 3 — Fig S3 [file JCMM-26-2404-s004.png]

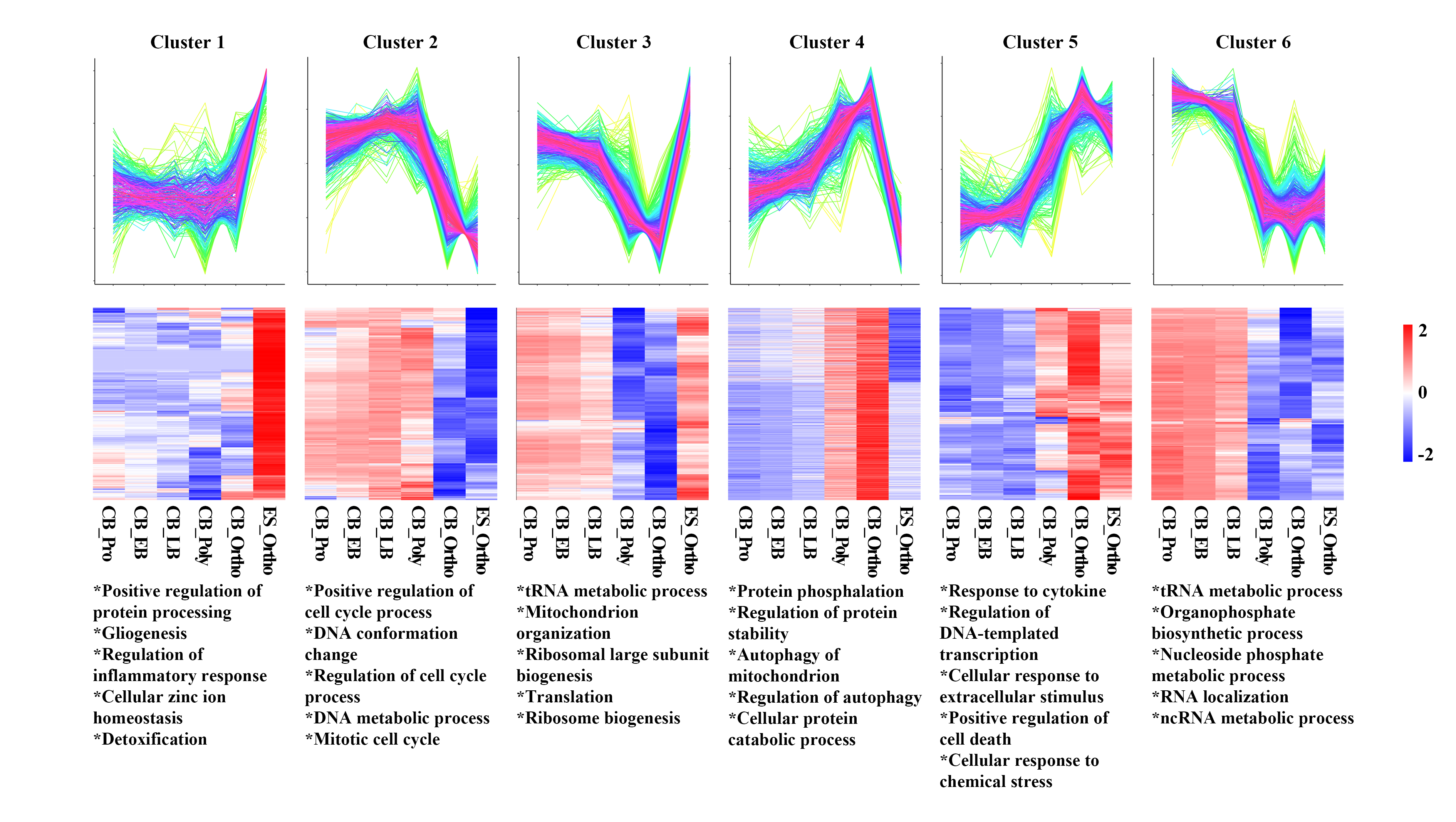

Supplement: Supplementary file 4 — Fig S4 [file JCMM-26-2404-s005.png]

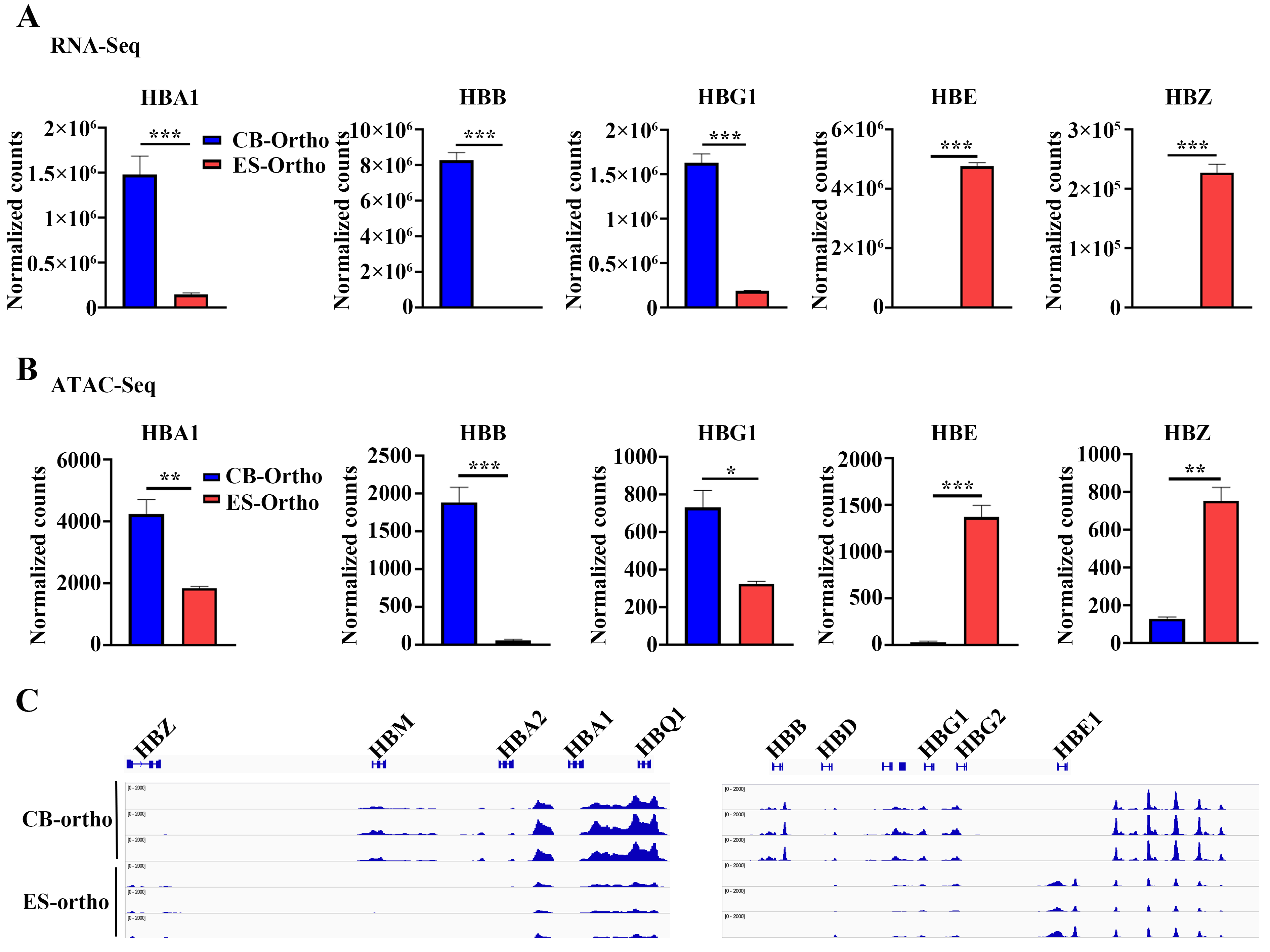

Supplement: Supplementary file 5 — Fig S5 [file JCMM-26-2404-s007.png]
